# The Impact of Hyperosmolality on Activation and Differentiation of B Lymphoid Cells

**DOI:** 10.3389/fimmu.2019.00828

**Published:** 2019-04-18

**Authors:** Ljiljana Cvetkovic, Stojan Perisic, Jens Titze, Hans-Martin Jäck, Wolfgang Schuh

**Affiliations:** ^1^Division of Molecular Immunology, Department of Internal Medicine III, Nikolaus-Fiebiger-Center, Friedrich-Alexander-University of Erlangen-Nürnberg, Erlangen, Germany; ^2^Department of Nephrology and Hypertension, Friedrich-Alexander-University Erlangen-Nürnberg, Erlangen, Germany; ^3^Department of Cellular Biophysics, Max Planck Institute for Medical Research, Heidelberg, Germany; ^4^Cardiovascular and Metabolic Disorders Program, Duke-NUS Medical School, Singapore, Singapore

**Keywords:** NaCl, hypertonicity, hyperosmolality, B cells, plasmablasts, plasma cells

## Abstract

B lymphocytes, as a central part of adaptive immune responses, have the ability to fight against an almost unlimited numbers of pathogens. Impairment of B cell development, activation and differentiation to antibody secreting plasma cells can lead to malignancy, allergy, autoimmunity and immunodeficiency. However, the impact of environmental factors, such as hyperosmolality or osmotic stress caused by varying salt concentrations in different lymphoid organs, on these processes is not well-understood. Here, we report that B cells respond to osmotic stress in a biphasic manner. Initially, increased osmolality boosted B cell activation and differentiation as shown by an untimely downregulation of Pax5 as well as upregulation of CD138. However, in the second phase, we observed an increase in cell death and impaired plasmablast differentiation. Osmotic stress resulted in impaired class switch to IgG1, inhibition of phosphorylation of p38 mitogen-activated kinase and a delayed NFAT5 response. Overall, these findings demonstrate the importance of microenvironmental hyperosmolality and osmotic stress caused by NaCl for B cell activation and differentiation.

## Introduction

B cell development in bone marrow of mice and men is a highly regulated, multi-step process which results in generation of B-cell-receptor-expressing immature B cells. Immature B cells leave bone marrow via blood stream and home to secondary lymphoid organs, in which they differentiate into naive, mature B cells. Lymph and blood perfuse secondary lymphoid organs, such as spleen, lymph nodes, tonsils, Peyer's patches and other gut-associated lymphoid tissues (GALT) and enable thereby the circulation of mature B cells through the body (1–3). Formation of germinal centers requires B cell migration, within secondary lymphatic organs (4, 5). It has been reported that skin (6), inflamed tissues (7, 8) and lymphoid organs (9) are the sites of increased osmolality compared to blood. Therefore, when trafficking through the body and changing, thereby, their microenvironment, immune cells are facing osmotic stress.

Macrophages, for instance, are attracted to the skin interstitium where they regulate Na^+^ release from the skin Na^+^ reservoir through lymph vessels driven by a NFAT5/VEGF-C signaling pathway (10). NFAT5 is the transcription factor responsible for hypertonicity-induced transcription of osmoadaptive genes, such as aldose reductase, neuropathy target esterase, betaine transporter, sodium/myo-inositol cotransporter, and sodium/chloride-dependent taurine transporter (11). Moreover, high salt diet can induce a pathogenic Th17 phenotype in mice (12) and thereby contributes to the development of EAE, a murine model of multiple sclerosis (13). Hybridoma cells increase antibody production when cultured under hyperosmotic conditions (14–16). Partial loss of NFAT5 function in NFAT5 heterozygous mice impairs B cell proliferation and reduces antigen specific responses to protein antigen (9). In addition, it has been reported that guanine nucleotide exchange factor Brx induces expression of NFAT5 through the activity of p38/MAPK in response to osmotic stress which enables B cells to differentiate and produce antibodies (17).

How increased osmolality affects B cell activation is poorly understood. Therefore, we wanted to investigate how osmotic stress influences B cell growth and viability and whether increased osmolality influences terminal B cell differentiation and antibody production. Therefore, we established an *in vitro* system for B cell cultivation under increased osmolality. To induce osmotic stress we used cell culture media with an increased NaCl concentration (+40 mM) in order to mimic an elevation in NaCl concentration similar to that found in the skin of rodents fed on a prolonged high salt diet (10) or in the infected skin of mice bitten by their cage mates (7), compared to the concentrations found in blood.

Here, we demonstrate that changes in osmolality affect B cell activation. LPS-stimulated B cells respond to increased osmolality in a biphasic manner. In the first phase, increased osmolality enhances differentiation into antibody-producing plasma cells; in the second phase, the initial boost disappears and we observed an arrest of proliferation and increased cell death. In contrast to other immune cells (T cells and macrophages), p38/MAPK pathway in B cells is inhibited by an increase in osmolality, moreover, an upregulation of NFAT5 does not seem to be regulated by this pathway. This *in vitro* model provides an excellent starting point to understand the molecular circuits that control B cell homeostasis under hyperosmotic conditions.

## Materials and Methods

### Mice

C57BL/6NRj mice were purchased from Janvier Labs (Le Genest Saint Isle, France). Blimp1-GFP mice were kindly provided by Steven Nutt (WEHI Institute, Australia). All animals were kept under pathogen-free conditions in the animal facility of the Franz-Penzoldt Center or Nikolaus-Fiebiger Center (Erlangen, Germany). All animal experiments were performed according to institutional and national guidelines.

### B Cell Isolation and Cell Culture

Naive B cells from the spleen were isolated by negative selection using the EasySep™ Mouse B cell Isolation Kit from StemCell Technologies (Vancouver, Canada). Previously obtained single cell suspensions were treated according to manufacturer's instructions. Briefly, cells were incubated with normal rat serum and EasySep™ Mouse B cell Isolation Cocktail at room temperature for 2.5 min. Later on, cells were labeled with the EasySep™ Streptavidin RapidSpheres™ for 2.5 min at room temperature. Using the EasySep™ Magnet, B cells were separated. Cell numbers were calculated and isolation purity was checked by flow cytometry. Cells were cultured in complete RPMI medium [containing 10% FCS, 1 mM sodium pyruvate, 50 U/ml penicillin, 50 μg/ml streptomycin, and 50 μM β-mercapto-ethanol (Gibco by Thermo Fisher Scientific, Waltham, MA, USA)] or complete RPMI medium supplemented with 40 mM NaCl to achieve hyperosmotic environment and activated with 10 μg/ml lipopolysaccharides (LPS; Sigma Aldrich, St. Louis, MO, USA). To induce class switch to IgG1 100 U/ml IL4 (Miltenyi Biosciences, Bergisch-Gladbach, Germany) was combined with 10 μg/ml LPS. Starting cell density was 0.25 × 10^6^ cells/ml.

### Antibodies and Flow Cytometric Analyses

For surface staining, 10^6^ isolated cells were stained with the respective antibodies for 20 min on ice. Unspecific bindings were blocked using CD16/CD32-unlabeled antibodies for 15 min on ice before each staining. For PAX5 intracellular staining, cells were fixed, permeabilized using the Foxp3 transcription factor staining kit (eBioScience, San Diego, CA, USA), and then stained as described. For measurements of phosphorylated p38 (p-p38) cells were fixed with 1.5% PFA and permeabilized with methanol and stained for 30 min at room temperature with anti-p-p38 (eBioscience, clone: ANIT4KK). AnnexinV was purchased from eBioscience, and staining was performed according to the manufacturer's protocol. Propidium iodide (PI) was added prior analysis. Fluorochrome-conjugated goat anti-mouse IgM (μHC specific) was obtained from Southern Biotechnology (Birmingham, AL, USA), and fluorochrome-conjugated monoclonal antibodies against CD19 (clone: 6D5), TACI (clone: ebio8F10-3), CD138 (clone: 281-2), CD62L (clone: MEL-14), CD69 (clone: H1.2F3), CD83 (clone: Michel-19), CD86 (clone: GL-1), PAX5 (clone: 1H9), IgG1 (clone: X56) were obtained from eBioscience, BD Biosciences, or BioLegend (San Diego, CA, USA). For analyses of surface markers and Blimp1:GFP expression we excluded doublets and gated on living cells according to FSC/SSC characteristics (for gating strategy see Supplementary Figure 1). For AnnexinV/PI staining no living cell gate was applied. Flow-cytometric data were collected on a Gallios flow cytometer (Beckman Coulter) and raw data was analyzed using either FlowJo (Ashland, OR, USA) or Kaluza (Beckman Coulter, Krefeld, Germany) software.

### CFSE Labeling

Intracellular and cell-surface proteins of B lymphocytes were CFSE-labeled (Sigma Aldrich, St. Louis, MO, USA) for cell division-tracking experiments. Cell suspensions of 20 × 10^6^ cells/ml in pre-warmed PBS were incubated with 5 μM CFSE for 15 min at 37°C. To stop labeling, equal volume of cold PBS was added. For efflux, cells were incubated in PBS at 37°C for 20 min. Cells were washed 3 times with PBS. Labeled cells were cultured in 0.25 × 10^6^ cells/ml density. Analysis of fluorescence signals was carried out using a Gallios flow cytometer (Beckman Coulter, Krefeld, Germany) and data was analyzed with FlowJo software (Ashland, OR, USA).

### RNA Isolation and q-Real Time PCR

LPS-blasts from 48 to 72 h cultures were homogenized using QIAShredder columns (Qiagen, Hilden, Germany). Total RNA was isolated from the homogenized lysate with the RNeasy Mini Kit (Qiagen) according to the manufacturer's instructions. RNA was reverse transcribed into cDNA using the Revert Aid First Strand cDNA synthesis kit (Thermo Scientific, Waltham, MA, USA) according to the manufacturer's instructions. qRT-PCR was performed using the SYBR™ Green PCR Master Mix (Applied Biosystems, Foster City, CA, USA) with gene-specific primers for NFAT5 (for: CAGCCAAAAGGGAACTGGAG, rev: GAAAGCCTTGCTGTGTTCTG). HPRT was used to control for integrity and abundance of the input RNA (for: TCAGTCAACGGGGGACATAAA, rev: GGGGCTGTACTGCTTAACCAG). Each template was measured in 2 technical replicates. Water and RNA control was used to determine if any contamination is present. Relative expression of the target genes was calculated according to the ΔΔCT method.

### Statistical Analysis

Significances and p-values were determined with IBM SPSS software (Armonk, NY, USA) using the Mann–Whitney U test. Significance is shown as ^*^p < 0.05, ^**^p < 0.01, and ^***^p < 0.001.

## Results

To induce osmotic stress in cell culture NaCl was used. 40 mM increase of NaCl concentration in the cell culture medium corresponds to the increased local concentration found in the skin of rodents fed on a prolonged high-salt diet (10) or in the infected skin of mice bitten by cage mates (7), relative to the concentrations maintained in blood. In the following part, standard cell culture conditions will be referred to as “normal salt”, whereas cell culture condition with NaCl added will be referred to as “high salt”.

**Figure 1:**
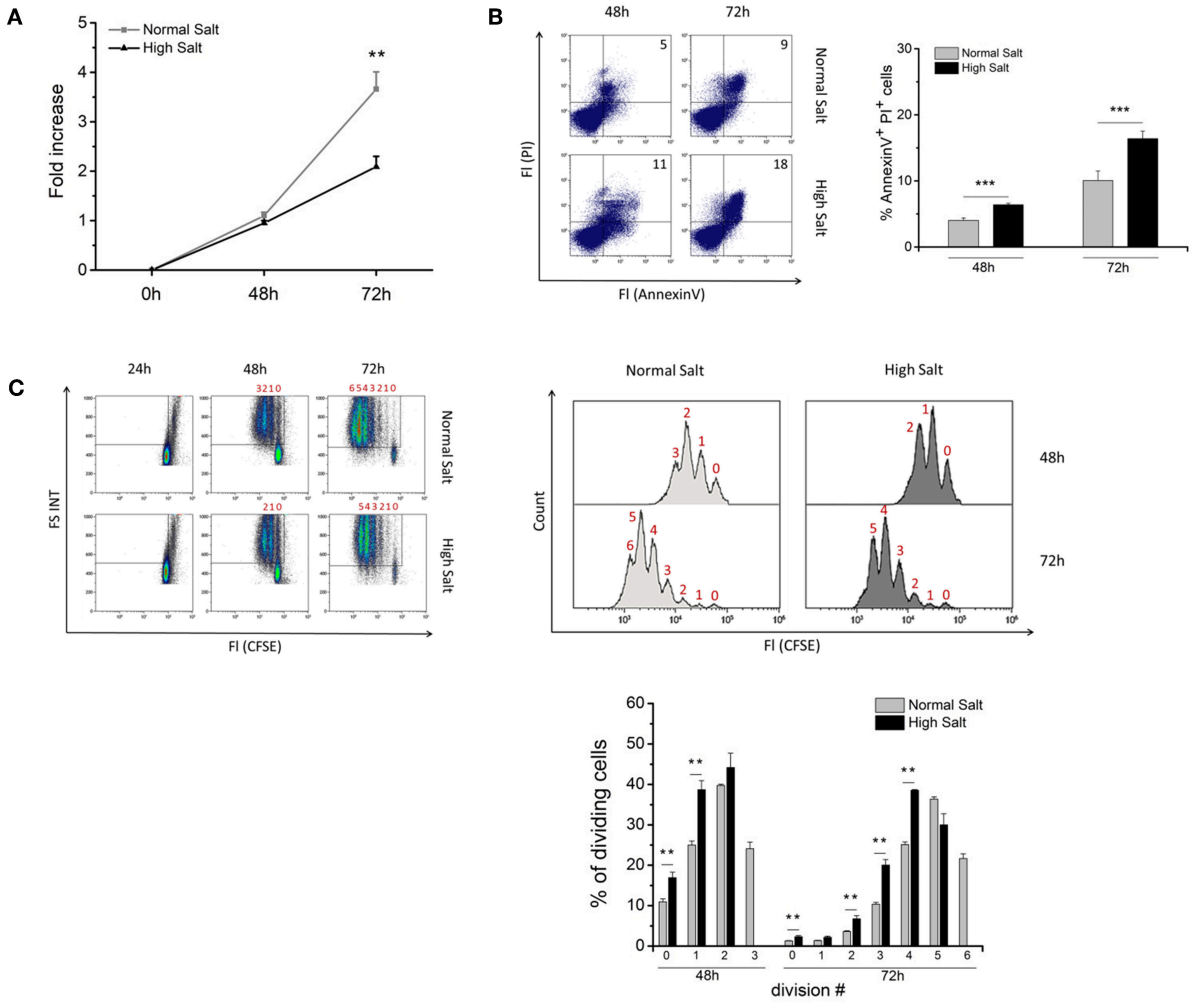
Effect of increased NaCl concentrations on growth, viability and proliferation of LPS-activated splenic B cells. Freshly isolated splenic B cells were cultured in normal medium (Normal Salt) and medium supplemented with an additional 40 mM NaCl (High Salt). Cells were activated with 10 μg/ml lipopolysaccharide (LPS). **(A)** Cell growth is presented by fold increase of cell numbers, which were calculated as the fold change between the starting cell density and cell density after indicated time points. The results show the summary of two independent experiments with a total of nine independent B cell isolates (*N* = 2, *n* = 9). **(B)** Viability of the cell culture was determined with AnnexinV/PI staining by flow cytometry. Frequencies of AnnexinV/PI positive cells are shown on representative plots for indicated time points. The summary of two independent experiments with a total of nine independent B cell isolates is presented as bar diagrams (*N* = 2, *n* = 9). **(C)** B cells were labeled with 5 μM CFSE and cultured for 24, 48, and 72 h in medium without and with the addition of 40 mM NaCl. Loss of CFSE fluorescence was analyzed by flow cytometry. The results are a summary of one experiment with a total of five independent B cell isolates (*N* = 1, *n* = 5). Data are presented as the mean ± SEM. **p ≤ 0.01; ***p ≤ 0.001, Mann-Whitney test.

To investigate whether an increase in osmolality has an impact on growth and viability of LPS-activated mouse B cells, splenic B cells from C57BL/6 WT mice were sorted by negative magnetic cell separation and cultured in the presence or absence of high salt conditions (adding 40 mM additional NaCl) over a period of 72 h. After 48 h, no difference in cell growth was observed between normal and high salt cultures (Figure 1A). However, on 72 h, cells cultured under high salt conditions were expanded only 2-fold, whereas cells under normal salt conditions were expanded 4-fold. Flow cytometric analysis of cell viability using propidium iodide (PI) in combination with Annexin V revealed that additional 40 mM NaCl significantly decreased the viability of the cells at all analyzed time points (Figure 1B).

Next, we investigated whether an increase in NaCl concentration affects the proliferation of LPS-activated B cells. Therefore, we labeled B cells with the division tracker dye CFSE and cultured them for 24, 48, and 72 h *in vitro* in presence or absence of additional 40 mM NaCl. Loss of CFSE fluorescence and the number of cell divisions were analyzed after 48 and 72 h. Compared to normal salt controls, B cells in high salt cultures showed an accumulation in cell division phases 0 and 1 at 48 h and in phases 2, 3, and 4 at 72 h, respectively. Moreover, high salt-treated B cells do not reach the maximum number of cell divisions achieved by B cells in normal cell cultures indicating that proliferation is impaired or delayed by high salt (Figure 1C). These findings clearly demonstrate that a hypertonic environment has a negative impact on B cell proliferation and cell viability.

**Figure 2:**
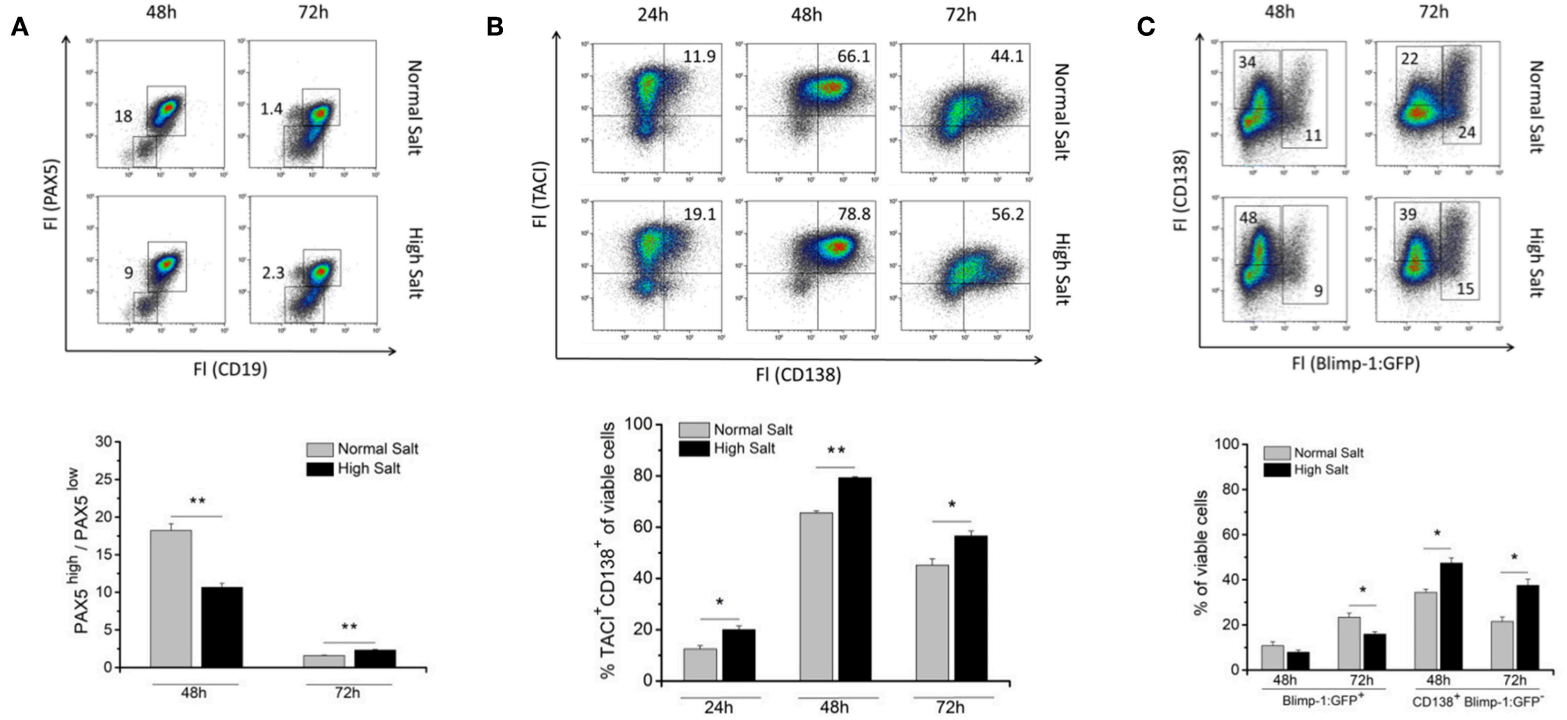
Effect of increased NaCl concentrations on B cell differentiation into plasmablasts. Freshly isolated splenic B cells were cultured with 10 μg/ml LPS with (High Salt) or without (Normal Salt) additional 40 mM NaCl. **(A)** Intracellular abundance of PAX5 was analyzed by flow cytometry. Results were presented as ratio of frequencies of PAX5^high^ and PAX5^low^ cells at indicated time points. Summary of one experiment with a total of five independent B cell isolates is presented as bar diagrams (*N* = 1, *n* = 5). **(B)** At indicated time points, the surface abundance of TACI and CD138 was analyzed by flow cytometry, and frequencies of TACI/CD138 positive cells were calculated. The results are a summary of three independent experiments with a total of thirteen independent B cell isolates (*N* = 3, *n* = 13). **(C)** Cells from Blimp-1:GFP reporter mouse were surface-stained with an antibody against CD138 and analyzed by flow cytometry at indicated time points. Two different populations according to their CD138 expression and GFP fluorescence are indicated as: Blimp-1:GFP^+^ (Blimp-1:GFP single positive cells and Blimp-1:GFP/CD138 positive cells) and CD138^+^ Blimp-1:GFP^−^ (CD138 single positive cells). Frequencies of cells are shown in representative dot plots. The results are a summary of three independent experiments with a total of eight independent B cell isolates (*N* = 3, *n* = 8). Data are presented as the mean ± SEM. *p ≤ 0.05; **p ≤ 0.01; Mann-Whitney test.

To assess whether an increase in osmolality in the cell culture medium affects B cell differentiation into antibody-secreting plasmablasts, freshly MACS-sorted splenic B cells were cultured with or without the addition of NaCl. 48 and 72 h later, cultures were analyzed for the presence of B cell identity markers PAX5 and CD19 (Figure 2A). PAX5 is a transcription factor known to be a master regulator of genes required to keep B cell identity (18, 19). PAX5 blocks plasma cell differentiation and must, therefore, be downregulated during the differentiation into plasma cells (PC) (20). According to the fluorescence intensities of PAX5 and CD19, two populations were distinguishable: PAX5^high^/CD19^high^ B cells and PAX5^low^/CD19^low^ plasmablasts (Figure 2A). Forty-eight hours upon LPS stimulation, the PAX5^high^/PAX5^low^ ratio (average 9) was significantly shifted in favor of PAX5^low^ cells in high salt cultures compared to the ratio found in normal salt cultures (average 19). This indicates that LPS-activated B cells differentiate faster into PAX5^low^ plasmablasts (Figure 2A). Interestingly, in 72 h LPS cultures, the ratios of PAX5^high^/PAX5^low^ observed on 48 h were reversed, i.e., the high salt culture contained fewer differentiated cells compared to the normal salt culture. The change in relative PAX5 abundance between 48 and 72 h suggests that salt initially boosts the B cell response and that plasma cell differentiation is strongly enhanced.

In addition, we analyzed the effect of high salt on the induction and abundance of common B cell activation markers such as CD62L, CD69, CD83, and CD86 at 6, 12, 24, 48, and 72 h after activation with LPS (Supplementary Figure 2A). We found a significant lower abundance of surface CD86 on B cells cultured under high salt conditions within the first 24 h after stimulation compared to normal salt cultures. However, at later time points no difference was observed. For CD62L, CD69, and CD83 only minor differences between normal and high salt cultures could be observed (Supplementary Figure 2A).

Next, differentiation of LPS-activated B cells in presence or absence of additional NaCl to TACI^+^/CD138^+^ plasmablasts was examined by flow cytometry (Figure 2B). TACI (transmembrane activator and CAML interactor and one of the BAFF receptors) and CD138 (syndecan-1, transmembrane heparin sulfate proteoglycan) were used as plasmablast markers (21). The frequencies of TACI^+^/CD138^+^ plasmablasts were significantly higher in 24, 48, and 72 h high salt cultures compared to cultures without additional NaCl (Figure 2B and Supplementary Figure 2B). These results indicate that B cells activated in a hypertonic environment differentiate faster to TACI^+^/CD138^+^ plasmablasts compared to an environment with lower salt concentration.

To further investigate the differentiation and activation of B cells challenged by increased NaCl concentration in the cell culture medium, intracellular abundance of Blimp-1, the master regulator of terminal B cell differentiation of activated B cells to the PCs and CD138 was analyzed (Figure 2C). For that purpose, B cells derived from the spleens of Blimp-1:GFP reporter mice was used (22). Cells activated *in vitro* display a notable heterogeneity in the expression of Blimp-1 and CD138, therefore, we distinguished two populations based on their expression of Blimp-1:GFP and CD138. Blimp-1:GFP^+^ cells: represent antibody secreting cells (22) and CD138^+^/Blimp-1:GFP^−^ presumably pre-plasmablasts. The most striking difference was a significant increase in the frequencies of CD138^+^/Blimp-1:GFP^−^ cells in high salt cultures after 48 and 72 h (Figure 2C). Frequencies of Blimp-1:GFP^+^ cells were, however, significantly reduced in high salt cultures after 72 h. In summary, these findings support again the hypothesis that a hyperosmolar environment fosters differentiation of activated B cells into antibody-secreting plasma cells. However, if plasma cells are retained in a high salt environment, they will die faster. Therefore, prolonged high salt environment acts as a potent negative regulator of plasma cell differentiation.

**Figure 3:**
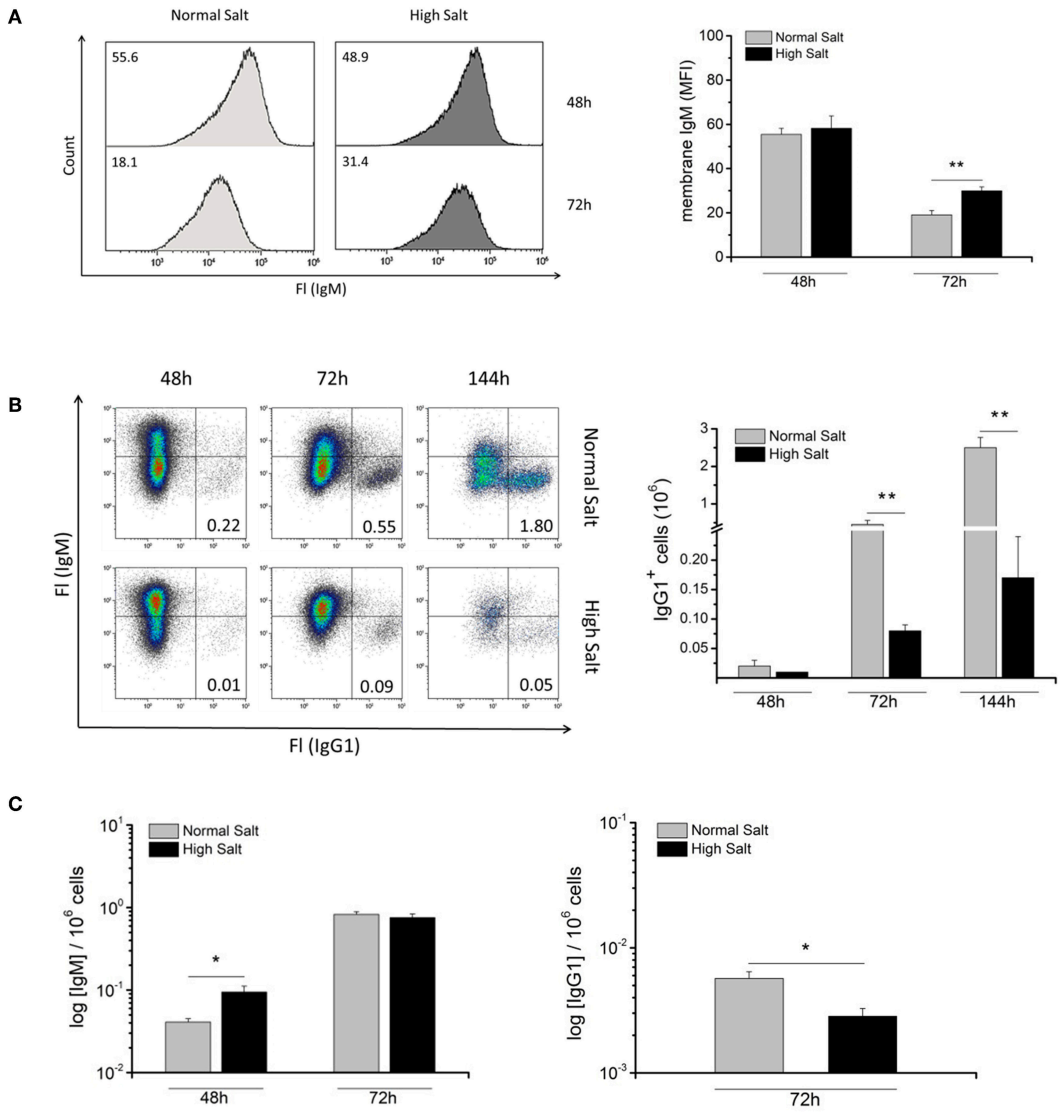
Effect of increased NaCl concentrations on abundance of membrane bound IgM, IgH class switch recombination and antibody production in LPS blasts. **(A)** Abundance of membrane IgM on LPS-activated B cells with (HS) or without (NS) additional NaCl was analyzed by flow cytometry at indicated time points. Representative histograms show mean fluorescence intensity (MFI) of membrane IgM. The results are a summary of two independent experiments with a total of eight independent B cell isolates (*N* = 2, *n* = 8). **(B)** Freshly isolated B cells were cultured in the absence (Normal Salt) or presence (High Salt) of additional NaCl and activated with 10 μg/ml LPS and, 100 U/ml IL4 to induce switching to IgG1. After 48, 72, and 144 h, flow cytometric analysis of surface IgM and IgG1 was used to detect switched cells. Data was presented as frequency of switched cells per million cells. The results show the summary of two independent experiments with a total of five B cell isolates (*N* = 2, *n* = 5). **(C)** Cell culture supernatant was collected and concentration of IgM (LPS) and IgG1 (LPS + IL4) was determined by Enzyme-linked Immunosorbent Assay (ELISA). The results are a summary of two independent experiments with a total of five independent B cell isolates (*N* = 2, *n* = 5). Data are presented as the mean ± SEM. *p ≤ 0.05; **p ≤ 0.01; Mann-Whitney test.

Upon primary antigen activation, naive B cells usually increase the production of the secreted form of IgM, which results in a decrease in the abundance of surface IgM. To determine whether hyperosmolality caused by NaCl changes the abundance of membrane-bound IgM on LPS-activated blasts, surface expression of IgM was analyzed in normal and high salt culture medium 48 and 72 h after LPS activation by flow cytometry (Figure 3A). After 48 h in culture, IgM surface abundance was the same under both culture conditions. However, after 72 h in culture there was a marked decrease in surface expression of IgM under normal salt conditions (MFI = 20) compared to high salt conditions (MFI = 30). These data apparently contradict the previous findings, which suggest that high salt initially favors plasma cell differentiation, i.e., surface IgM was expected to disappear faster in high salt cultures. Hence, high surface abundance of IgM after 72 h suggests that less IgM is secreted, which is in line with our previous finding that high salt cultures contain less Blimp-1^+^ antibody-secreting plasmablasts.

To determine whether a change in salt concentrations affects the ability of LPS blasts to switch their Ig class from IgM to other isotypes, such as IgG1, normal and high salt cultures were supplemented with LPS and IL4 and analyzed for the presence of membrane IgG1 for 48, 72, and 144 h (Figure 3B). Frequencies of IgG1^+^ B cells in high salt cultures were decreased at all analyzed time points, with significant differences at 72 and 144 h compared to normal salt cultures. Under normal salt conditions, the number of membrane IgG1^+^ cells after 72 h was ~500,000, while in the high salt culture there was significantly less IgG1^+^ cells, i.e., merely 80,000 cells. These results indicate that an increase in osmolality impairs IgH class switch recombination (CSR) and limits, therefore, the Ig effector repertoire.

To assess the effect of increasing osmolality on antibody-secretion, supernatants from normal salt and high salt cultures were collected and analyzed for the concentration of secreted Igs by ELISA (Figure 3C). The amounts of secreted Igs per cell were calculated on the basis of total secreted amount of antibodies and numbers of live cells in cultures. After 48 h, cells in high salt cultures secreted significantly more secreted IgM per cell than cells under normal salt conditions, which was in line with increase in TACI^+^/CD138^+^ plasmablasts observed by flow cytometry in high salt cultures. However, after 72 h, the amount of secreted IgM per cell in normal and high salt cultures was comparable, indicating that longer exposure to high salt conditions leads to cell death of antibody-secreting plasmablasts. Moreover, the normal salt culture contained significantly more secreted IgG1 per cell after 72 h (Figure 3C). This reduction of IgG1 secretion under high salt conditions is in line with the class switch defect observed by flow cytometry in high salt cultures. In summary, time limited, short, exposure to high salt conditions favors B cell differentiation and antibody production, but impairs class switch recombination.

**Figure 4:**
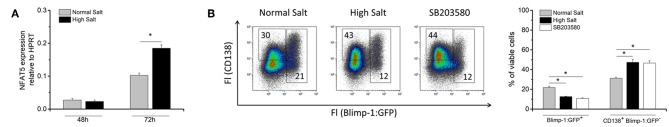
Effect of increased NaCl concentrations on NFAT5 expression and p38/MAPK phosphorylation in activated mouse B cells. **(A)** Cells from 48 to 72 h of LPS cell culture in presence (High Salt) or absence (Normal Salt) of additional NaCl were harvested and RNA was isolated. After cDNA reaction, real-time qPCR was performed to determine expression of NFAT5 in given samples. Results present NFAT5 expression relative to HPRT (average ± SD). Four independent measurements were performed in three technical replicates for each sample. **(B)** After 72 h, LPS activated B cells from the Blimp-1:GFP reporter mouse in the presence (High Salt) or absence (Normal Salt) of additional NaCl and SB203580 were analyzed by flow cytometry for their CD138 and GFP expression. Two different populations according to their CD138 expression and GFP fluorescence are indicated as: Blimp-1:GFP^+^ (Blimp-1:GFP single positive cells and Blimp-1:GFP/CD138 positive cells) and CD138^+^ Blimp-1:GFP^−^ (CD138 single positive cells). Frequency of cells was shown on representative dot plots. The results show the summary of two independent experiments with a total of five independent B cell isolates (*N* = 2, *n* = 5). Data are presented as the mean ± SEM. *p ≤ 0.05; Mann-Whitney test.

Hyperosmotic stress can activate p38, a mitogen-activated protein kinase (MAPK) upstream of NFAT5 and it is, therefore, involved in hypertonic activation of NFAT5 (23). It was shown that increased salt concentration enhances p38-dependent NFAT5 activation in CD4^+^ T cells and macrophages (7, 12). To assess whether an increase in osmolality in the cell culture medium upregulates NFAT5 expression in B cells, NFAT5 transcript abundance was analyzed by real-time PCR analysis in LPS blasts. Signals were analyzed by the ΔΔCT method and normalized to HPRT (Figure 4A). Relative NFAT5 expression after 48 h in culture under normal salt and high salt conditions was comparable. However, we observed an 8-fold and 4-fold increase in relative NFAT5 signals in high and normal salt after 72 h of culture, respectively. The 2-fold increase of NFAT5 expression in high salt cultures compared to normal salt cultures was significant. These results suggest that the impact of salt on LPS-induced NFAT5 expression occurs later in plasmablast differentiation.

Furthermore, we analyzed whether p38 phosphorylation (p-p38) changed in B cells cultured in a normal salt or a high salt medium in the presence of LPS for 10 and 20 min by flow cytometry (Supplementary Figure 3). Normal salt cultures served as a positive control for the staining of a monoclonal antibody recognizing the phosphorylation of p38 at threonine residue T180 and tyrosine residue Y182. After 10 min, the mean fluorescence intensities (MFI) for phosphorylated p38 (p-p38) in normal salt cultures showed an increase from 250 in untreated cells to a MFI of 300; in contrast decrease of MFI from 250 to 220 was detected in high salt cultures. After 20 min, a significant difference in p-p38 fluorescence signal intensities between normal and high salt cultures was observed. At this time point, the intensity of the p-p38 signal in high salt cultures was even lower (MFI = 200) than the intensity measured after 10 min suggesting that high salt inhibits p38 phosphorylation and therefore, its activation. Increase in osmolality resulted in an inhibition of p38 phosphorylation and, therefore, activation and downstream signaling pathway in B cells.

To confirm that high salt indeed inhibits p38 activation, we used SB203580, a chemical inhibitor of p38/MAPK (24) and cultured freshly isolated splenic B cells from Blimp-1:GFP reporter mice 72 hours with LPS under normal salt and high salt conditions and in the presence of the SB203580 (Figure 4B). As previously shown, increased NaCl concentration led to accumulation of CD138^+^/Blimp-1:GFP^−^ cells, but did not favor differentiation into Blimp-1^+^ antibody-secreting cells (Figure 2C). As expected, inhibitor-treated cultures showed a comparable phenotype in regards to Blimp-1:GFP and CD138 staining as seen in high salt cultures, i.e., the frequency of CD138^+^/Blimp-1:GFP^−^ cells increased, while frequencies of Blimp-1:GFP^+^ cells were reduced in inhibitor-treated cultures to the same extend as they were in high salt cultures.

These results indicate that salt induces the inhibition of p-p38 activation, which might explain the inefficient differentiation into Blimp-1^+^ antibody-secreting cells under the high salt culture conditions.

## Discussion

Previous findings by Go and colleagues that lymphoid tissues are hyperosmotic compared to blood (9) established the basis for addressing osmoadaptive mechanisms in lymphocytes. The impact of environmental factors, such as hyperosmolality or osmotic stress caused by differences in salt concentrations in different lymphoid organs, on B cell activation is not well-understood. Here, we performed an *in vitro* study with the aim to elucidate the effect of increased osmolality caused by NaCl on B cell survival, activation and differentiation and demonstrated that B cell differentiation under increased osmolality can be divided into two phases (Supplementary Figure 4). In phase I the increase of osmolality boosts their activation and differentiation as demonstrated by PAX5 downregulation and corresponding CD138 upregulation. In phase II a stark decrease in cell growth is observed, which correspond to the increased cell death and the inhibition of proliferation. Cells from this point on decelerate their differentiation with more PAX5^+^ and less Blimp-1^+^ cells.

LPS-stimulated B cells undergo a massive proliferation and differentiation to antibody-secreting plasmablasts (25–27). Using a CFSE-based proliferation assay we demonstrated that increased NaCl leads to an accumulation of cells in cell division phases 0 and 1 after 48 h and phases 2, 3, and 4 after 72 h. High salt treated B cells do not reach the maximum number of cell divisions achieved by B cells in normal cell cultures indicating that proliferation is impaired or delayed by high salt. In parallel, we could confirm a reduction of cell growth and viability in primary splenic mouse B cells upon treatment with an additional +40 mM NaCl, which has been previously reported in different cell types, renal medullary cells (28), lymphocytes (29), thymocytes (30) and DT40 chicken B cells (31). Our study suggest that as osmotic stress persists, a substantial DNA damage caused by additional NaCl arrests the DNA reparation mechanism, which results in apoptosis of differentiated plasmablasts (32–35).

Using Blimp-1:GFP reporter mice an increase in the frequencies of CD138^+^/Blimp-1^−^ cells was noted, which are presumably pre-plasmablasts (22). Their increase is independent of Blimp-1 expression (36), which suggests that NaCl enhances and fosters the initial activation signaling in B cells. This notion is strongly supported by a primary decrease of PAX5^+^ cells and upregulation of CD138 expression. Therefore, increased NaCl concentrations in B cell cultures boost the initial activation signals. We showed that osmotically stressed cells show high expression of membrane IgM, and an impaired class switch to IgG1. A initiation of CSR and AID induction depends on reduction of PI3K activity (37). Therefore, we speculate that excessive NaCl concentration increases PI3K activity upon B cell activation. This induces a regulatory signal through PI3K, possibly by suppressing the AID function and thus, suppressing CSR. It is also interesting to mention that PI3K signaling is necessary for p53-dependent induction by DNA damage (38, 39). PI3K-dependent p53 activity is therefore one potential mechanism for B cell response under osmotic stress.

Several publications in which hybridoma cells were cultured under increased osmolality presented evidence of increased antibody production (14–16). Along this line, our results demonstrated that primary LPS-activated B cells, cultured for a shorter period of time, produced significantly more IgM (Figure 3C).

Osmotic stress can activate mitogen-activated protein kinase (MAPK) p38 (23, 40) and is likely to play an important role in NFAT5 activation, acting as an upstream regulator in various cells, such as Jurkat cells (17), macrophages (7) and primary T cells (12). On the other hand, it has been shown that NFAT5 heterozygous mice (NFAT5^+/Δ^) have impaired antibody responses and reduced B cell proliferation in hypertonic medium (9). Here we demonstrate that B cells show a delayed upregulation of NFAT5 transcription as a response to osmotic stress compared to macrophages (10) and T cells (12). Go and colleagues demonstrated that osmolality in tissues such as spleen or thymus is elevated compared to blood. This implies that isolated B cells from the spleen already came from a hyperosmotic environment and were adapted to such conditions, while, contrary to them, newly generated plasmablasts are more sensitive to increased osmolality and therefore in higher demand for osmoadaptation. That assumption would explain the delayed NFAT5 upregulation observed in our experiments, resulting from increased NFAT5 expression in newly generated plasmablasts. However, this hypothesis needs to be confirmed using more sensitive techniques capable of measuring osmolality within different tissue compartments (i.e., germinal centers, B and T cell zone) and discriminating between intravascular and extravascular (interstitial) tissue fluid.

Contrary to the data published (23, 40), we found that high salt inhibits p38 signaling in B cells. Hence, normal salt B cell cultures activated p38, i.e., p38 has been phosphorylated upon LPS stimulation (41). That implies that LPS-dependent p38 activation is inhibited by NaCl. The chemical inhibition using SB203580 confirmed our conclusion: a decrease in antibody-secreting cells and an increase in CD138^+^ cells. Our results demonstrate that NFAT5 signaling in B cells is not dependent on p38 activation. This suggests that in osmotically stressed B cells, NFAT5 activity is, in contrast to T cells and macrophages, regulated by p38 independent mechanisms.

In summary, short term increase in osmolality fosters B cell activation and differentiation, but chronical exposure to NaCl dampens the initial boost of activation and differentiation and results in augmented cell death of differentiated cells. A deeper understanding of salt-dependent effects on B cell responses will be important to elucidate the functional role of micro-environmental salt in lymphoid tissues on B cell activation and the impact of dietary salt on adaptive immune responses, B cells malignancies and autoimmune diseases.

## Ethics Statement

This study was carried out in accordance with the German Law on Care and Use of Laboratory Animals. Euthanasia and organ preparation were approved by the local authorities (Landratsamt Erlangen-Hoechstadt, Erlangen, Germany).

## Author Contributions

LC designed the study, planned, and performed experiments, analyzed and interpreted data and wrote the manuscript. SP designed the study, interpreted data, and edited the manuscript. JT initiated the study and supported the work. H-MJ made key suggestions, assisted in the design of experiments, interpreted data and critically reviewed the manuscript. WS designed the study, planned experiments, analyzed and interpreted data and wrote and finalized the manuscript.

### Conflict of Interest Statement

The authors declare that the research was conducted in the absence of any commercial or financial relationships that could be construed as a potential conflict of interest.
