# Supplementary material for: The Impact of Hyperosmolality on Activation and Differentiation of B Lymphoid Cells
Source: Front Immunol. 2019 Apr 18;10:828. doi: 10.3389/fimmu.2019.00828 (PMC6482216; doi:10.3389/fimmu.2019.00828)

Supplementary Material

The impact of hyperosmolality on activation and differentiation of B lymphoid cells

Ljiljana Cvetkovic, Stojan Perisic, Jens Titze, Hans-Martin Jäck and Wolfgang Schuh*

*** Correspondence:** Dr. Wolfgang Schuh (wolfgang.schuh@uk-erlangen.de)

1 Supplementary Data

Supplementary Figure 1: Gating strategy. Single cells were discriminated according to their forward scatter characteristics (FSC int versus FSC peak), and viable cells were identified according to their forward scatter (FSC) and sideward scatter (SSC) characteristics. Single, viable cells were used as an input gate for further gating and defining cell populations.

Supplementary Figure 2: Effect of increased NaCl concentrations on (A) CD62L, CD69, CD83, CD86 and (B) TACI, CD138 surface abundance. Splenic, freshly isolated B cells were stimulated for 6, 12, 24, 48 and 72 hours with 10 µg/ml LPS in normal medium (Normal Salt) and medium with increased NaCl concentration (High Salt). At indicated time points, the surface abundance of CD62L, CD69, CD83 and CD86 was analyzed by flow cytometry, and frequencies of $TACI^+$, $CD138^+$, $CD62L^+$ and $CD83^+$ cells or MFIs of CD69 and CD86 were calculated. The results show the summary of one experiment with a total of four B cell isolates (N=1, n=5). Data are presented as the mean ± SEM. *$p\leq0.05$; **$p\leq0.01$; ***$p\leq0.001$, Mann-Whitney test.

Supplementary Figure 3: Effect of increased NaCl concentrations on p38/MAPK phosphorylation in activated mouse B cells. Splenic, freshly isolated B cells were stimulated for 10 and 20 min with 10 µg/ml LPS in normal medium (Normal Salt; NS) and medium with increased NaCl concentration (High Salt; HS). Flow cytometry was used for analysis of p-p38 MFI. The results

show the summary of two independent experiments with a total of six independent B cell isolates (N=2, n=6). Data are presented as the mean ± SEM. *$p \leq 0.05$; **$p \leq 0.01$; ***$p \leq 0.001$, Mann-Whitney test.

Supplementary Figure 4: Biphasic effect of elevated NaCl concentrations on LPS-mediated B cell activation, proliferation and differentiation.

Supplementary Figure 1

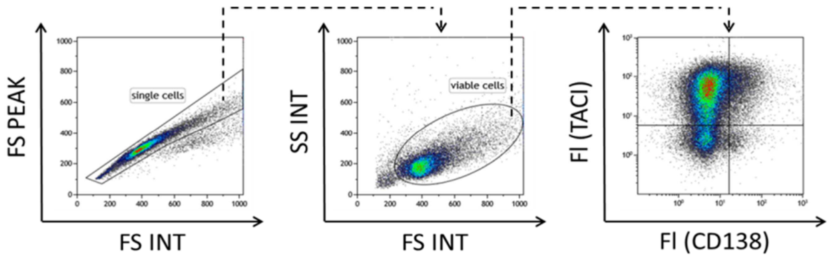

Supplementary Figure 2A

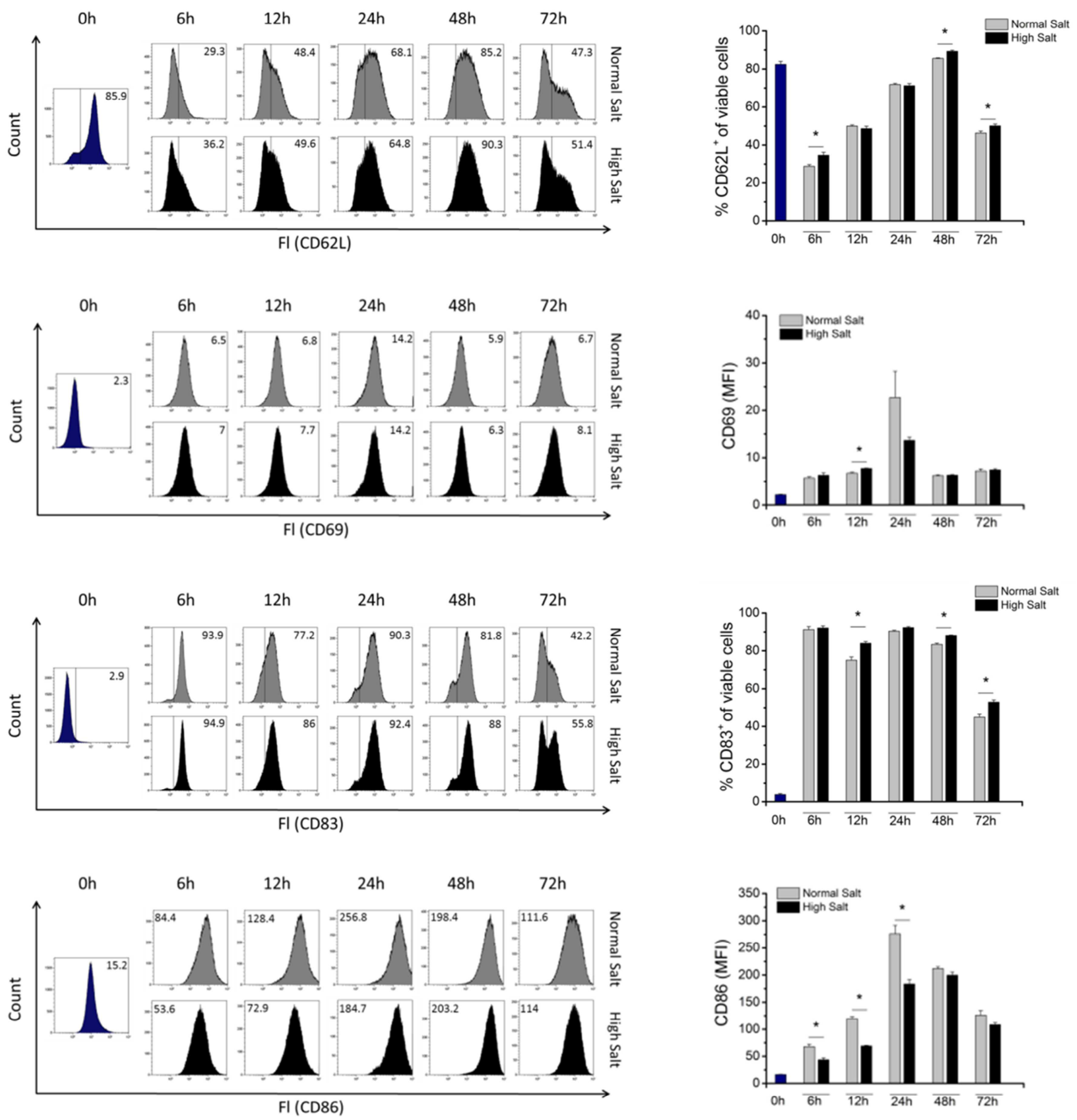

Supplementary Figure 2B

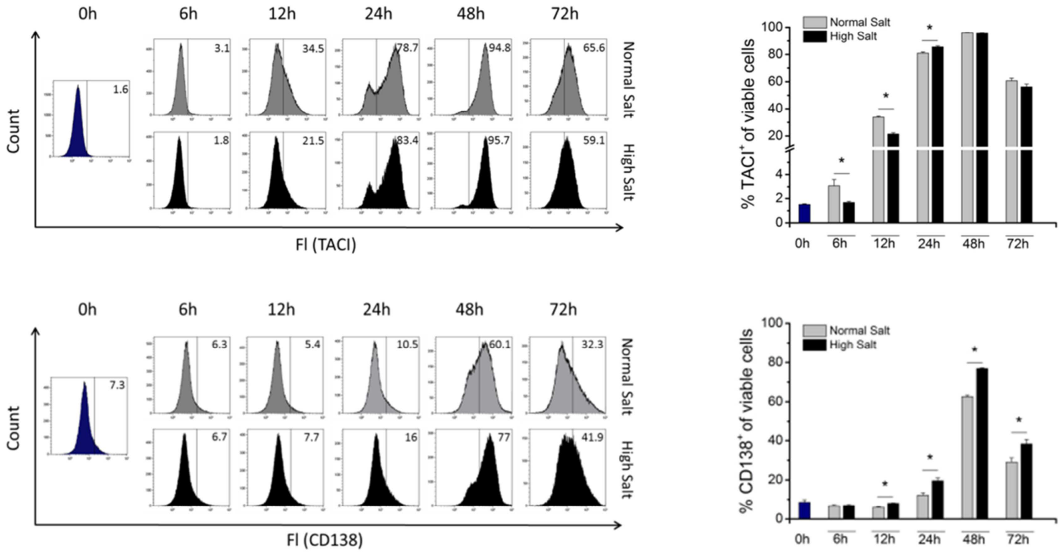

Supplementary Figure 3

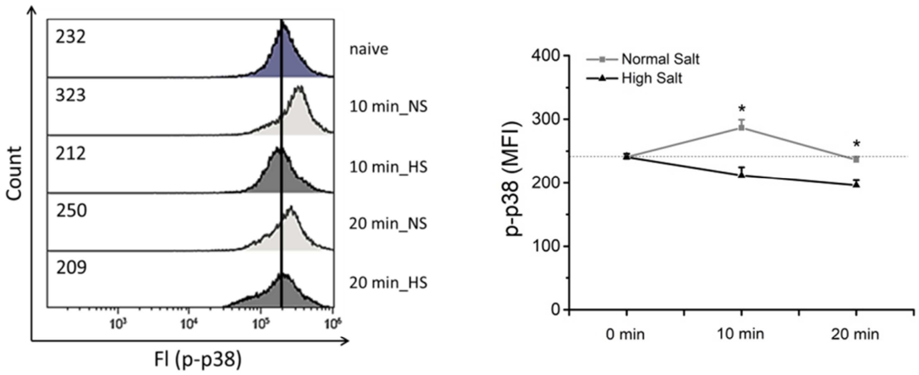

Supplementary Figure 4

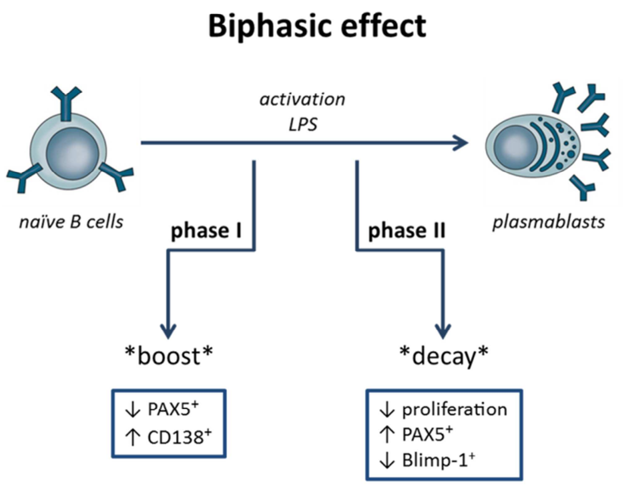

Supplement: Supplementary file 1 [file Data_Sheet_1.pdf]
